# No Evidence that *CD33* rs12459419 Polymorphism Predicts Gemtuzumab Ozogamicin Response in Consolidation Treatment of Acute Myeloid Leukemia Patients: Experience of the PETHEMA Group

**DOI:** 10.1155/2022/3132941

**Published:** 2022-08-23

**Authors:** Tamara Castaño-Bonilla, Eva Barragán, Claudia Sargas, Alejandro Sanz, Lorenzo Algarra, Pilar Herrera-Puente, Raimundo García-Boyero, Manuel Barrios, David Martinez-Cuadron, Rebeca Rodriguez-Veiga, Blanca Boluda, Cristina Gil, Josefina Serrano-López, Joaquín Martínez-López, María José Sayas-Lloris, María Teresa Olave, Rosalía Riaza-Grau, Teresa Bernal-Del Castillo, María José Larrayoz, Raquel Amigo, Antonio Jiménez-Velasco, Joaquín Sánchez, Rosa Ayala, Carlos Blas, Daniel Lainez, Juana Serrano-López, Miguel A. Sanz, Juan M. Alonso-Domínguez, Pau Montesinos

**Affiliations:** ^1^Hematology Department, Hospital Universitario Fundación Jiménez Díaz, Madrid, Spain; ^2^Instituto de Investigación Sanitaria (IIS-FJD), Hospital Universitario Fundación Jiménez Díaz, Madrid, Spain; ^3^Hematology Department, Hospital Universitari i Politècnic La Fe, Valencia, Spain; ^4^Hematology Department, Hospital HM Sanchinarro, Madrid, Spain; ^5^Hematology Department, Hospital General Universitario de Albacete, Albacete, Spain; ^6^Hematology Department, Hospital Universitario Ramón y Cajal, Madrid, Spain; ^7^Hematology Department, Hospital General de Castellón, Castellón de la Plana, Spain; ^8^Hematology Department, Hospital Regional Universitario de Málaga, Málaga, Spain; ^9^Hematology Department, Hospital General Universitario de Alicante, Alicante, Spain; ^10^Hematology Department, Hospital Universitario Reina Sofía, Córdoba, Spain; ^11^Hematology Department, Hospital Universitario Doce de Octubre, Madrid, Spain; ^12^Hematology Department, Hospital Universitario Doctor Peset, Valencia, Spain; ^13^Hematology Department, Hospital Clínico Universitario Lozano Blesa, Zaragoza, Spain; ^14^Hematology Department, Hospital Universitario Severo Ochoa, Madrid, Spain; ^15^Hematology Department, Hospital Universitario Central de Asturias, Oviedo, Spain; ^16^Molecular Biology Department, Cimalab Diagnosis, Clínica Universitaria de Navarra, Pamplona, Spain; ^17^Biobanco La Fe, Instituto de Investigación Sanitaria La Fe, Valencia, Spain

## Abstract

Gemtuzumab ozogamicin (GO) is a conjugate of a monoclonal antibody and calicheamicin, which has been reapproved for the treatment of acute myeloid leukemia (AML). AML patients with the *CD33* rs12459419 CC genotype might benefit from the addition of GO to intensive treatment in contrast to patients with CT/TT genotypes. Nevertheless, contradictory results have been reported. We sought to shed light on the prediction of GO response in AML patients with rs12459419 polymorphism who were treated with GO in the consolidation (*n* = 70) or reinduction (*n* = 20) phase. The frequency distribution of the rs12459419 polymorphism in the complete cohort of patients was 44.4% (*n* = 40), 50% (*n* = 45), and 5.6% (*n* = 5) for CC, CT, and TT genotypes, respectively. Regarding the patients treated with GO for consolidation, we performed a Kaplan-Meier analysis of overall survival and relapse-free survival according to the rs12459419 polymorphism (CC vs. CT/TT patients) and genetic risk using the European Leukemia Net (ELN) 2010 risk score. We also carried out a Cox regression analysis for the prediction of overall survival, with age and ELN 2010 as covariates. We found no statistical significance in the univariate or multivariate analysis. Additionally, we performed a global Kaplan-Meier analysis for the patients treated with GO for reinduction and did not find significant differences; however, our cohort was too small to draw any conclusion from this analysis. The use of GO in consolidation treatment is included in the approval of the compound; however, evidence regarding its efficacy in this setting is lacking. Rs12459419 polymorphism could help in the selection of patients who might benefit from GO. Regrettably, in our cohort, the rs12459419 polymorphism does not seem to be an adequate tool for the selection of patients who might benefit from the addition of GO in consolidation cycles.

## 1. Introduction

Acute myeloid leukemia (AML) is a heterogeneous disorder characterized by a clonal expansion of myeloid progenitors [1]. AML remains a difficult-to-treat disease, and novel efficacious therapies are needed. Immunotherapeutic strategies have been proved highly effective in other hematological malignancies [2]. Gemtuzumab ozogamicin (GO) is a humanized anti-CD33 IgG4 mAb conjugated to a cytotoxic agent N-acetyl gamma calicheamicin. GO targets the membrane antigen CD33 that is present on the majority of AML blasts [3]. The efficacy of the drug in three open-label phase II trials resulted in an accelerated approval in 2000 by the US Food and Drug Administration [4]. However, a large follow-up study (SWOG-S0106) showed an increase in early death with the use of GO in AML patients, so the drug manufacturer voluntarily withdrew the US New Drug Application in 2010 [5]. In 2014, a meta-analysis of five randomized controlled trials [6–10] showed that adding GO to induction chemotherapy improved overall survival (OS) and relapse-free survival (RFS) in favorable-risk and, to a lesser degree, intermediate-risk AML patients. Based on these results, GO was reapproved. Nevertheless, three of the trials of the meta-analysis also included GO in consolidation cycles, and the possible clinical benefit of adding GO in the consolidation cycle has not been clearly elucidated.

GO treatment has been associated with hepatotoxicity and hepatic veno-occlusive disease/sinusoidal obstruction syndrome (VOD/SOS), myelosuppression, bleeding/thrombocytopenia, infusion-related reactions, and tumor lysis syndrome. In the ALFHA 701 study, drug-induced neutropenia was more prolonged in the GO group than in the control group after each cycle of consolidation. In addition, the median time to recovery of platelets was longer for patients in the GO arm than in the control arm for each treatment course (persistent grades III-IV thrombocytopenia was observed in 20% of patients in the GO group vs. 2% in the control group) [11].

**Figure 1:**
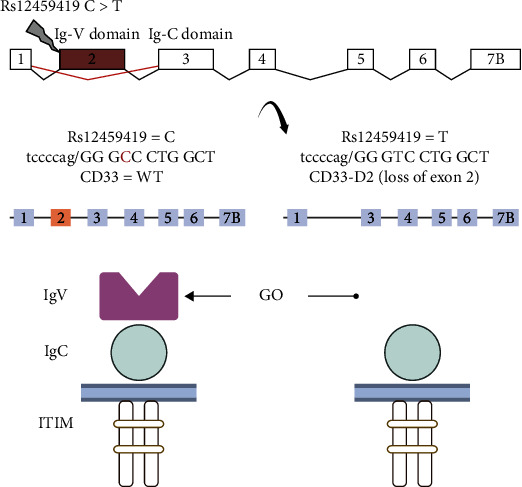
Mechanism of action of GO. Abbreviations: GO: gemtuzumab ozogamicin.

Because GO is ineffective in many patients and/or may have adverse effects, there is interest in understanding favorable and unfavorable prognostic factors. Therefore, it is important to further refine the selection of patients that might achieve a favorable response from GO administration. Two studies conducted in pediatric and *NPM1*-mutated AML adult patients treated with GO during the induction phase suggested a potential value of *CD33* genotype as a predictor of response [12, 13]. However, the gathered data from several trials did not validate the results described previously [14, 15]. Specifically, subjects with the *CD33* rs12459419 CC genotype (about 50% of study entrants) seemed to have a significantly lower risk of relapse and better event-free survival and disease-free survival after GO therapy, whereas this benefit was not seen in patients with the CT or TT genotypes. Rs12459419:c.41C > T; p.Ala14Val is related to the loss of exon 2 in the *CD33* transcript, which results in a shorter isoform of the CD33 protein product (D2-CD33) that lacks the IgV domain. This domain, which is encoded by exon 2, is recognized by the antibody that is conjugated to calicheamicin in GO [16–19]. Consequently, the loss of this domain can interfere with the clinical efficacy of GO (Figure 1).

In the present study, we analyzed whether *CD33* rs12459419 polymorphism influences the clinical outcome in adult AML patients treated with GO during consolidation or reinduction cycles.

## 2. Material and Methods

### 2.1. Patients and Samples

The PETHEMA AML epidemiologic registry (NCT02607059) includes the data of patients diagnosed with AML, regardless of the treatment administered. The primary patient and disease characteristics were collected retrospectively (Table 1). We reviewed the prospectively collected data of 106 adult AML patients treated with GO from the PETHEMA AML epidemiologic registry following the PETHEMA 2007 protocol. Pretreatment samples were available for *CD33* polymorphism determination in 90 patients: 48 men and 42 women. The median age was 55.3 years (range 17.4–76.9 years) in these patients with newly diagnosed AML between 2005 and 2013. Seventy patients received GO 3 mg/m (2) in the consolidation cycle and 20 in reinduction treatment. Finally, 19 patients underwent autologous hematopoietic stem-cell transplantation (HSCT), and 33 patients went through allogeneic HSCT.

### 2.2. CD33 SNP Screening

We received DNA (*n* = 85) and RNA (*n* = 14) samples from seven centralized PETHEMA laboratories. The samples analyzed in this study were collected at the diagnostic stage. DNA was extracted using automated or manual DNA extraction kits (Qiagen, Hilden, Germany) following the manufacturer's recommendations. DNA quantification was done using NanoDrop (ThermoFisher Scientific, Waltham, MA) or Qubit fluorometer (ThermoFisher Scientific, Waltham, MA) [20]. Samples were screened for the *CD33* SNP using HaeIII restriction enzyme digestion of polymerase chain reaction (PCR)-generated amplicons. The cDNA was synthesized using the High-Capacity cDNA Reverse Transcription kit (ThermoFisher, Vilnius, Lithuania). PCR products of 266 base pairs (bp) and 204 bp were generated from genomic DNA and cDNA, respectively, using HotStarTaq® DNA polymerase (Qiagen, Germany) using the manufacturer's recommended conditions and primers (*CD33* exon 1/F: 5′-­-CTGGAAGCTGCTTCCTCAGACATG-­-3′; *CD33* exon 2/R: 5′­-GAACCAGTAACCATGAACTGGGGAGTT-­-3′) at an annealing temperature of 66°C. Products were digested overnight with HaeIII (New England Biolabs, Hitchin, UK) and separated on a 3% agarose gel to discriminate between the C allele (94 + 29 + 143 bp) and T allele (94 + 172 bp) using the DNA and C allele (61 + 143 bp) and T allele (204 bp) using the cDNA.

### 2.3. Statistical Analysis

OS was calculated from the date of diagnosis of AML until death in all included patients. RFS was calculated from the date of achieving complete response (CR) or CR with incomplete hematologic recovery (CRi) until the date of relapse or death due to any cause. CR and CRi were defined according to current 2017 ELN guidelines [21]. OS and RFS were estimated by the Kaplan-Meier method and compared by log-rank test. Group comparisons were defined according to the rs12459419 genotype (i.e., CC vs. CT and TT). Patients treated with GO in the consolidation phase were analyzed globally and in subgroups according to the ELN 2010 genetic risk [22] (intermediate I and II groups were merged). Multivariate Cox regression analysis was used for OS prediction, with age and ELN genetic risk model as covariates. All patients, who received GO as reinduction, were analyzed separately. A separate analysis using the ELN 2010 genetic risk was not performed due to the reduced number of patients in the reinduction group. Mann–Whitney test was used to compare time to platelet (>50.000/*μ*l) and neutrophil recovery (>500/*μ*l) among patients who received 3 + 7 plus GO as a consolidation cycle. A *P* value below 0.05 was considered statistically significant. SPSS version 19.0 (IBM, Arkmon) was used for the analyses.

## 3. Results

The frequency distribution of the rs12459419 genotypes in the whole patient population (*n* = 90) were 44.4%, 50%, and 5.6% for the CC, CT, and TT polymorphism, respectively. From the total of 70 patients treated with GO during the consolidation cycle, there were 32 patients (45.7%) with CC genotype and 38 (54.3%) with CT/TT genotype. The median duration of OS in the CC group was 2.7 years (95% confidence interval: 1.0-4.3) vs. 4.4 years (95% CI: 0.5-8.2) in the CT/TT group (*P* = 0.9). The median RFS in the CC group was 1.5 years (95% CI: 1.1-1.9) vs. 1.7 years (95% CI: 0.7-2.6) in the CT/TT group (*P* = 0.5).

**Figure 2:**
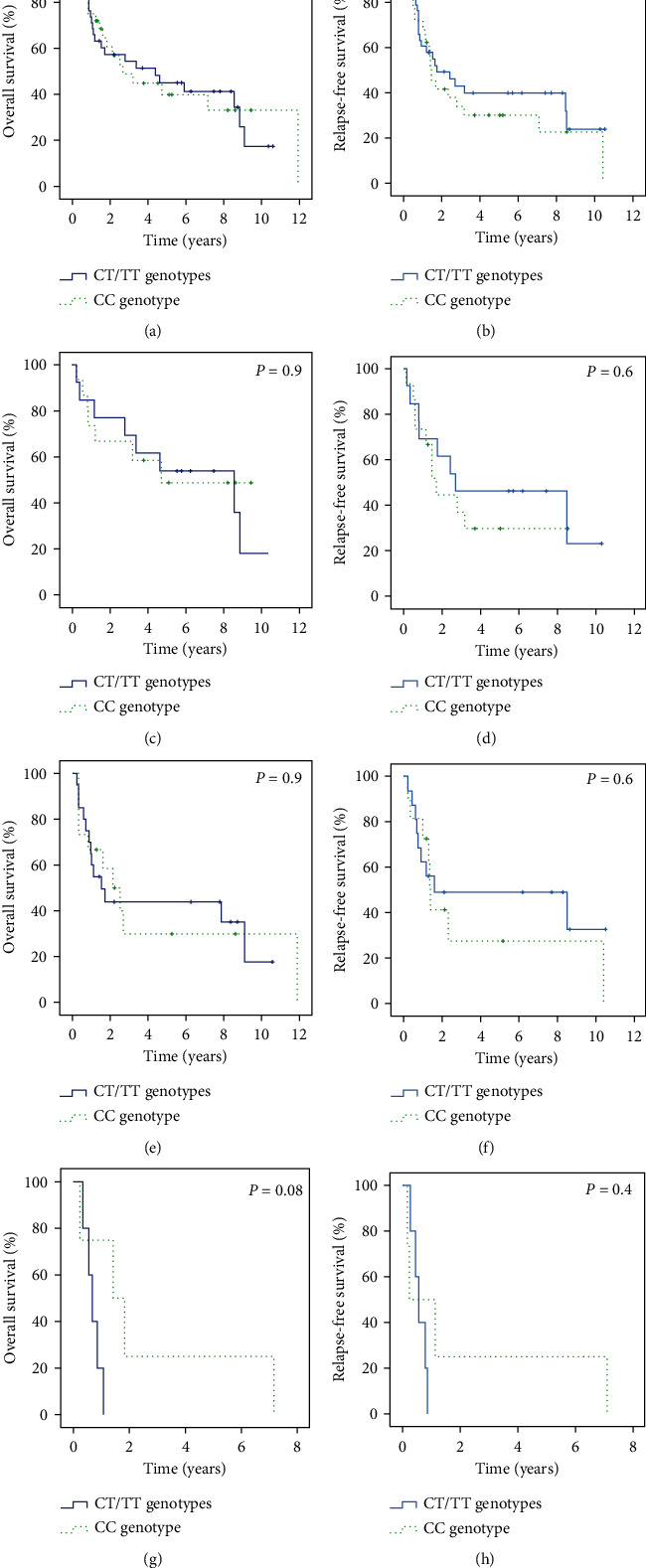
Clinical outcome stratified by the *CD33* rs12459419 polymorphism for patients treated with GO during the consolidation phase (a–h). (a) Overall survival (OS). (b) Relapse-free survival (RFS). (c–h) OS and RFS according to *CD33* rs12459419 polymorphism and genetic risk. (c and d) OS and RFS in the favorable group; (e and f) OS and RFS in the intermediate group; and (g and h) OS and RFS in the adverse group.

Furthermore, a separate analysis based on the ELN 2010 genetic risk [22] was performed, including 64 patients. In the analysis of OS and RFS, the distribution of rs12459419 genotype was as follows: 15 patients (23.4%) with CC genotype and 13 (20.3%) with CT/TT genotype in the favorable-risk group, 11 patients (17.2%) with CC genotype and 16 (25%) with CT/TT genotype in the intermediate-risk group, and four (6.3%) with CC genotype and five (7.8%) with CT/TT genotype in the adverse-risk group. The OS in the favorable group was 4.7 years (95% CI: not calculable [NC]) in CC genotype and 8.6 years (95% CI: 1.4-15.4) in CT/TT genotype (*P* = 0.9). The OS in the intermediate group was 2.5 years (95% CI: 1.6-3.5) in CC genotype and 1.7 years (95% CI: NC-7.8) in CT/TT genotype (*P* = 0.9). The OS in the adverse group was 0.8 years (95% CI: NC-3.0) in CC genotype and 0.1 years (95% CI: 0.4-0.9) in CT/TT genotype (*P* = 0.8). The RFS in the favorable group was 1.7 years (95% CI: 1.3-2.7) in CC genotype and 2.7 years (95% CI: NC-7.5) in CT/TT genotype (*P* = 0.6). The RFS in the intermediate group was 1.4 years (95% CI: 1.3-1.6) in CC genotype and 1.6 years (95% CI: NC-9.4) in CT/TT genotype (*P* = 0.6). The RFS in the adverse group was 0.2 years (95% CI: NC-1.2) in CC genotype and 0.6 years (95% CI: 0.3-0.8) in CT/TT genotype (*P* = 0.4) (Table 2 and Figure 2). Cox regression analysis for the prediction of OS showed that ELN 2010 and age were independent predictor factors (*P* = 0.03, HR: 1.4, [95% CI: 1.03-1.85] and *P* = 0.02, HR: 1.04, [95% CI: 1.01-1.07], respectively). Rs12459419 genotype was not an independent predictor factor (*P* = 0.5, HR: 0.8, [95% CI: 0.4-1.5]). Proportional hazard assumptions were checked by adding the interaction of every variable with time (Table 3).

Finally, in the 20 patients treated with GO during the reinduction phase, the distribution of rs12459419 genotype in OS analysis was 12 patients (60%) with CC genotype and eight (40%) with CT/TT genotype. The median duration of OS in the CC group was 0.6 years (95% CI: 0.5-0.8) vs. 0.3 years (95% CI: 0.3-0.3) in the CT/TT group (*P* = 0.3). The distribution of rs12459419 genotype in RFS analysis was nine patients (45%) with CC genotype and three (15%) with CT/TT genotype. The median duration of RFS in the CC group was 0.6 years (95% CI: 0.3-0.9) vs. 0.6 years (95% CI: 0.5-2.7) in the CT/TT group (*P* = 0.8). (Supplementary Material see available here).

The drug-induced neutropenia was similar in the CT/TT and CC genotype groups after the first cycle of consolidation with 3 + 7 plus GO: a median of 23 days (range 19-31) vs. 20 days (range 14-28), respectively (*P* = 0.3). In addition, we did not find statistical differences regarding the time to recovery of platelets: a median of 25.5 days (range 23-54) in the CT/TT group and 25.5 days (range 20-43) in the CC group (*P* = 0.8). No patient developed VOD/SOS during cycles of treatment prior to allogeneic HSCT. Regrettably, data on the incidence of VOD/SOS in the postallogeneic HSCT setting were not collected.

## 4. Discussion

Personalized medicine is based on selecting an adequate treatment for every individual patient [23]. We evaluated the impact of the *CD33* rs12459419 genotype on the efficacy of GO treatment in AML patients. Investigating a potential lack of clinical benefit of CT and TT genotypes of the polymorphism, we performed comparisons between the CT/TT and CC groups, who were supposed to benefit from GO treatment. Our real-life cohort is composed of 90 AML patients: none of them were included in clinical trials, and all were treated with GO in reinduction (*n* = 20) or consolidation treatment (*n* = 70) following the PETHEMA 2007 protocol. In our cohort, the rs12459419 *genotype* does not have a prognostic impact in terms of OS or RFS among AML patients treated with GO in either the consolidation or reinduction cycle. Additionally, the time to hematological recovery after the first consolidation cycle with 3 + 7 plus GO cycle was not influenced by the SNP genotype.

The clinical impact of the genotype of this splicing polymorphism in AML patients treated with GO-containing chemotherapy is controversial. In a study in a mainly pediatric population, but also including young adults (COG-AAML0531) with AML (0-29 years old), a benefit of GO was demonstrated in the induction treatment in those with CC genotype of *CD33* rs12459419 polymorphism [12]. In contrast to our results, a recent large study in adult AML patients who were eligible for intensive therapy and had an *NPM1* mutation showed improved RFS and reduced cumulative incidence of relapse in patients with CC genotype treated with GO during the induction phase [13]. Conversely, the British group analyzed a cohort of mainly adult AML patients (13-69 years old) and found that the patients who received GO in induction treatment did not achieve clinical improvement in terms of OS or RFS regardless of the rs12459419 genotype [14]. Moreover, as in our population, a study in patients treated with decitabine plus GO during the consolidation phase (*n* = 113) did not reveal a significant impact of the SNP on OS or RFS. However, this study was performed in a patient population unlikely to benefit from GO as it included high-risk myelodysplastic syndrome patients and AML patients with unfavorable risk features [15]. In contrast to the described studies, our cohort was composed of patients who received GO at either consolidation or reinduction cycles. Interestingly, three out of five trials that were included in the meta-analysis of GO efficacy administered GO also in consolidation cycles [10]. Nevertheless, only the MRC AML15 trial performed a new randomization process for consolidation. MRC AML15 trial did not show differences in consolidation depending on GO administration, but this study neither showed differences regarding GO administration in the induction cycle [7]. Indeed, GO approval includes induction and consolidation doses following the ALFA 0701 trial scheme protocol [9]. Therefore, the possible clinical benefit of adding GO only in the consolidation phase has not been clearly elucidated. Nevertheless, a high proportion of minimal residual disease eradication has recently been reported with the addition of GO to consolidation treatment with HIDAC [24]. This finding may support the clinical efficacy of GO in consolidation cycles. Besides, although GO addition to consolidation had not achieved clinical benefit in the overall population, GO could improve outcomes in patients with CC genotype of the *CD33* rs12459419 polymorphism. Regrettably, our study has shown that this polymorphism does not have prognostic value in this setting.

Our study has some limitations. First, our patients were selected from an observational registry, which can be interpreted as a limitation given the heterogeneity of treatment or as a strength because our data are more real-life than those derived from a clinical trial. Second, since the number of patients was small, it would be necessary to increase the sample size to validate our results. However, our results support the findings of the previously published study with the largest cohort [14, 15].

In the event that the genotype of the rs12459419 polymorphism played a role in the mechanism of action of GO, it would modify the effect of the drug on leukemia and normal hematopoiesis only in patients with CC genotype. We would expect a longer time to recovery of peripheral blood counts in these patients without an increase in this time in patients with CT or TT genotypes. In our cohort, we did not find differences in the recovery of neutrophils and platelets based on the SNP genotype. These results were similar to those recently published by Short et al., which support the lack of prognostic value of the rs12459419 polymorphism found in our cohort [1].

## 5. Conclusions

In summary, in our population of 20 AML patients treated with GO in reinduction, we found no prognostic significance of *CD33* rs12459419 polymorphism, but no conclusions can be drawn due to the reduced amount of patients analyzed. In the consolidation setting, we analyzed 70 patients and did not observe differences between groups. Therefore, *CD33* rs12459419 polymorphism does not seem to be an adequate tool to select patients who might benefit from the addition of GO in consolidation cycles.

## Figures and Tables

**Table 1:** Baseline characteristics of AML patients treated with GO 3 mg/m(2) during reinduction and consolidation phases based on CD33 *rs12459419* polymorphism.

*CD33* rs12459419 genotype in AML patients treated with GO in reinduction and consolidation phases						
	Reinduction treatment			Consolidation treatment		
Patients characteristics	Total; *n* =20	CC (*n* = 12; 60%)	CT/TT (*n* = 7/1; 35%/5%)	Total; *n* = 70	CC (*n* = 32; 46%)	CT/TT (*n* = 34/4; 49%/6%)
Median age at diagnosis, years	51	49.5	53.9	57.2	57.2	57.7
Range	17.4–64.2	17.4-64.1	30.4-61.2	24.8–76.9	24.8-70.0	28.2-76.9
Median follow-up, years	0.58	0.58	0.25	2.13	1.96	2.17
Sex						
Female	10 (50%)	8 (67%)	2 (25%)	36 (51%)	18 (56%)	18 (47%)
Male	10 (10%)	4 (33%)	6 (75%)	34 (49%)	14 (44%)	20 (53%)
Cytogenetic risk (ELN 2010)						
Favorable risk	5 (25%)	4 (33%)	1 (13%)	28 (40%)	15 (47%)	13 (34%)
Intermediate-I risk	2 (10%)	2 (17%)	─	20 (29%)	8 (25%)	12 (32%)
Intermediate-II risk	6 (30%)	3 (25%)	3 (38%)	7 (10%)	3 (9%)	4 (11%)
Adverse risk	6 (30%)	3 (25%)	3 (38%)	9 (13%)	4 (13%)	5 (13%)
Median leucocytes at diagnosis, 109/L	14.7	16.0	22.8	17.5	17.5	17.5
Range	1.6-206.6	1.6-100.5	1.9-206.6	0,7-379	1,1-379	0,7-324
Median hemoglobin at diagnosis, g/dl	8.7	8.7	8.8	9.5	9,5	9,5
Range	4.6-11.2	4.6-11.2	5.5-10.9	5-13,9	5-13,9	5,3-13,5
Median platelets at diagnosis, 109/L	53	53	49	65	65	62
Range	15-261	30-261	15-97	14-336	14-317	13-336
Treatment						
Induction therapy						
Idarubicin+cytarabine (3 + 7)	19 (95%)	11 (92%)	8 (100%)	59 (84%)	27 (84%)	32 (84%)
Idarubicin+cytarabine (2 + 5)	1 (5%)	1 (8%)	─	9 (13%)	5 (16%)	4 (11%)
Other treatments	─	─	─	2 (3%)	─	2 (5%%)
Reinduction						
3 + 7 with GO	1 (5%)	─	1 (12.5%)	─	─	─
IDA-FLAGO	19 (95%)	12 (100%)	7 (87.5%)	─	─	─
Other treatments	─	─	─	─	─	─
Consolidation						
HDARAC+GO	─	─	─	14 (20%)	6 (19%)	8 (21%)
3 + 7 + GO	─	─	─	56 (80%)	26 (81%)	30 (79%)
HDARAC	6 (30%)	4 (33%)	2 (25%)	─	─	
IDA-FLAG	2 (10%)	2 (17%)	─	─	─	
Support treatment	1 (5%)	1 (8%)	─	─	─	
Stem cell transplant						
Allogeneic hematopoietic cell transplantation	11 (55%)	8 (67%)	3 (38%)	22 (31%)	11 (34%)	11 (29%)
Autologous hematopoietic cell transplantation	─	─	─	19 (27%)	6 (19%)	13 (29%)

Abbreviations: GO: gemtuzumab ozogamicin; ELN: European Leukemia Net; IDA-FLAG/IDA-FLAGO: idarubicin, fludarabine, cytarabine, and G-CSF ± GO; ARAC (100 mg/m^2^ during 5 days); HDARAC (1-3 g/m^2^ during 1, 3, and 5 days of the cycle).

**(a):** 

Overall survival				
rs12459419 genotypes	Genetic risk (ELN 2010)	N	Median (95% CI)	*P*
CC	Global	32	2.7 y (1.0-4.3)	0.9
	Favorable risk	15	4.7 y (NC)	0.9
	Intermediate risk	11	2.5 y (1.6-3.5)	0.9
	Adverse risk	4	0.8 y (NC-3.0)	0.8
CT/TT	Global	38	4.4 y (0.5-8.2)	0.9
	Favorable risk	13	8.6 y (1.4-15.4)	0.9
	Intermediate risk	16	1.7 y (NC-7.8)	0.9
	Adverse risk	5	0.1 y (0.4-0.9)	0.8

**(b):** 

Relapse-free survival				
rs12459419 genotypes	Genetic risk (ELN 2010)	N	Median (95% CI)	*P*
CC	Global	32	1.5 y (1.1-1.9)	0.5
	Favorable risk	15	1.7 y (1.3-2.7)	0.6
	Intermediate risk	11	1.4 y (1.3-1.6)	0.6
	Adverse risk	4	0.2 y (NC-1.2)	0.4
CT/TT	Global	38	1.7 y (0.7-2.6)	0.5
	Favorable risk	13	2.7 y (NC-7.5)	0.6
	Intermediate risk	16	1.6 y (NC-9.4)	0.6
	Adverse risk	5	0.6 y (0.3-0.8)	0.4

Abbreviations: ELN: European Leukemia Net; CI: confidence interval; NC: not calculable.

**Table 3:** Cox proportional hazard regression model analysis of overall survival.

Overall survival		
Covariate	HR (95% CI)	*P*
Age	1.04 (1.01-1.07)	0.02
Genetic risk (ELN 2010)	1.4(1.03-1.85)	0.03
*CD33* genotypes	0.8 (0.4-1.5)	0.5

Abbreviations: ELN: European Leukemia; CI: confidence interval.

## Data Availability

Data are available upon request from the authors.

## Supplementary Materials

Supplementary Materials Supplement Table 1: Kaplan-Meier analysis of overall survival and relapse-free survival in patients treated with GO in the reinduction phase. Supplement Figure 1: clinical outcome stratified according to *CD33* rs12459419 polymorphism for patients treated with GO during the reinduction phase (a, b) (a) Overall survival. (b) Relapse-free survival.
