# Supplementary material for: No Evidence that CD33 rs12459419 Polymorphism Predicts Gemtuzumab Ozogamicin Response in Consolidation Treatment of Acute Myeloid Leukemia Patients: Experience of the PETHEMA Group
Source: Dis Markers. 2022 Aug 23;2022:3132941. doi: 10.1155/2022/3132941 (PMC9427256; doi:10.1155/2022/3132941)
Supplement: Supplementary Materials — Supplement Table 1: Kaplan-Meier analysis of overall survival and relapse-free survival in patients treated with GO in the reinduction phase. Supplement Figure 1: clinical outcome stratified according to CD33 rs12459419 polymorphism for patients treated with GO during the reinduction phase (a, b) (a) Overall survival. (b) Relapse-free survival. [file 3132941.f1.docx]

**Supplementary Material**

**Supplement Table 1.** Kaplan-Meier analysis of overall survival and relapse-free survival in patients treated with GO in the reinduction phase

| Overall survival | | | |
| --- | --- | --- | --- |
| rs12459419 genotypes | **N** | **Median (95% CI)** | ***P*** |
| CC | 12 | 0.6 y (0.5-0.8) | 0.3 |
| CT/TT | 8 | 0.3 y (0.3-0.3) | 0.3 |
| Relapse-Free Survival | | | |
| rs12459419 genotypes | **N** | **Median (95% CI)** | ***P*** |
| CC | 9 | 0.6 y (0.3-0.9) | 0.8 |
| CT/TT | 3 | 0.6 y (0.5-2.7) | 0.8 |

**
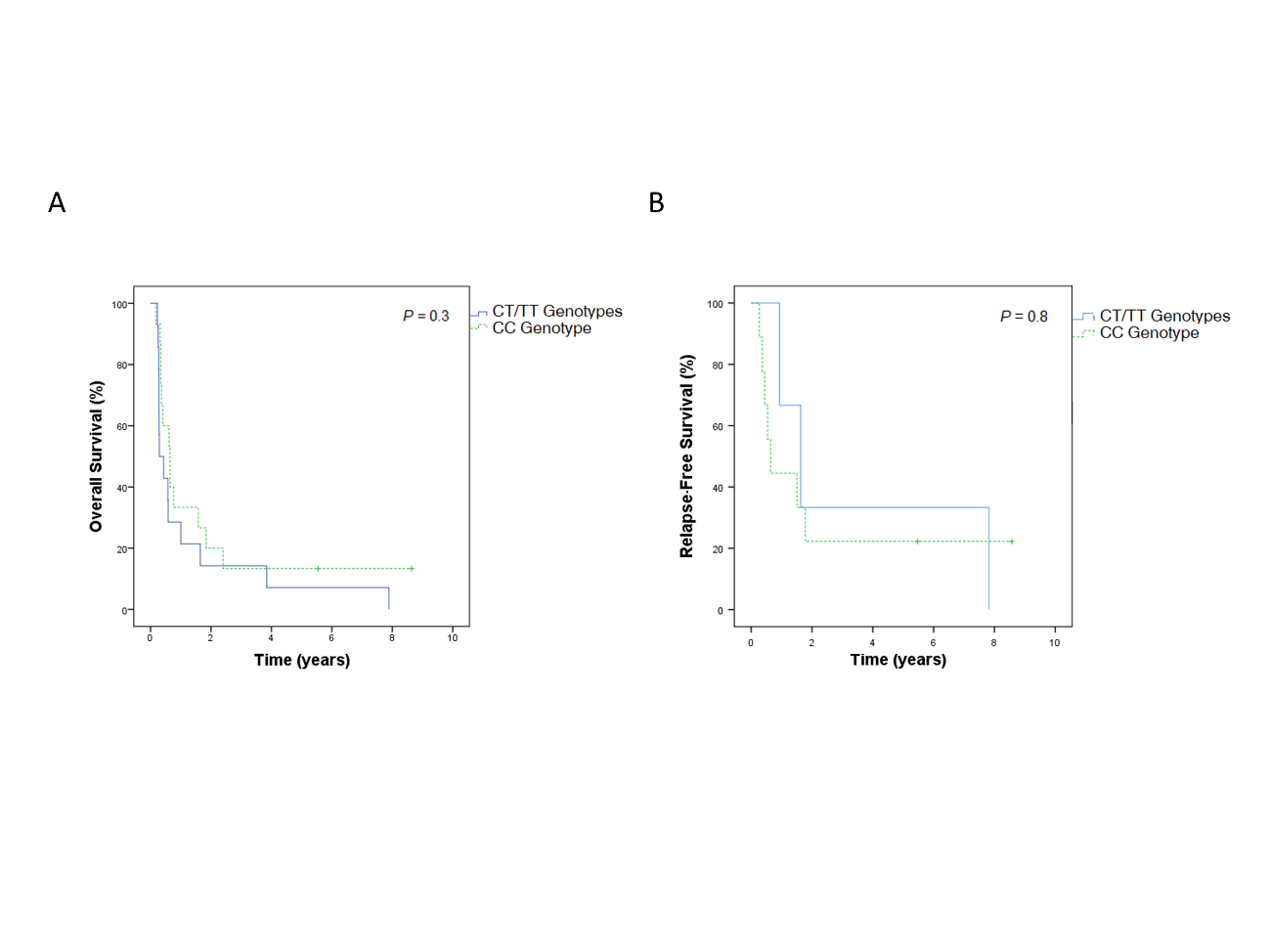
Supplement Fig. 1**: Clinical outcome stratified according to *CD33* rs12459419 polymorphism for patients treated with GO during the reinduction phase (A, B)

(A) Overall survival. (B) Relapse-free survival.
